# A novel approach to button battery removal in a two-and-half year-old patient’s esophagus after ingestion: a case report

**DOI:** 10.1186/s12887-022-03142-3

**Published:** 2022-02-17

**Authors:** Hung-Chun Wang, Shu-wei Hu, Ke Jian Lin, An-Chyi Chen

**Affiliations:** 1 Department of Pediatrics, Chang Bing Show Chwan Memorial Hospital, Lukang, Changhua Taiwan; 2 Department of Pediatrics, Tungs’ Taichung MetroHarbor Hospital, Taichung, Taiwan; 3 Department of Pediatric Gastroenterology of Children’s Hospital, China Medical University, Taichung, Taiwan

**Keywords:** Battery balloon extraction, Button battery ingestion, Button battery retrieval, Disc battery extraction, Flexible versus rigid endoscopy, Case report

## Abstract

**Background:**

Accidental swallowing of a foreign body occurs more frequently in children than in adults. Among these cases, button battery impaction in the esophagus may cause severe complications. While prevention is always ideal, if button battery impaction is suspected, immediate diagnosis and retrieval are important.

**Case presentation:**

We introduce a novel method for retrieval of a button battery after ingestion by a 2.5-year-old child. When the patient arrived at our center, the battery was incarcerated in the upper esophagus. The battery could not be removed, despite the use of several methods such as alligator forceps under endoscopy and net retrieval. We decided to use a novel method that combined endoscopic balloon extraction and forceps retrieval. This resulted in a push-and-pull effect, creating synergy and easy removal of the battery. There were no long term complications based on the follow-up endoscopy examination.

**Conclusions:**

This new procedure was very effective for removing the esophageal foreign body. When button battery in esophagus was too tight to be removed by the traditional retrieval methods, this procedure was suggested to use. It could be performed at medical institutions. If it fails or esophageal perforation (iatrogenic or spontaneous) occurs, pediatric surgeons could take over immediately.

## Background

Children are prone to accidentally swallowing button batteries, usually from products around the home [1]. The incidences of button battery ingestion (BBI) are most frequently attributed to children younger than 4 years old [1]. Younger children tend to have lithium battery (the same as "button battery") impaction in the esophagus rather than in the stomach [2, 3]. Button battery impaction in the esophagus is an emergency event that may lead to severe complications and even mortality. Nowadays, higher voltages and bigger-sized lithium batteries are made for powering toys [2, 3], resulting in more frequent and devastating complications from the 1980s up to 2010. And yet despite health education by media in many countries, the incidence rate of BBIs has not changed significantly [1, 3, 4].

Several methods for button battery removal have been introduced, including balloon extraction with fluoroscopy, magnetic tip oral-gastric tube extraction with fluoroscopy, flexible endoscopy, and rigid esophageal endoscopy. In some patients, the battery is very difficult to remove and a surgical approach is needed. Herein, we report on our novel method to remove a button battery that was incarcerated in the upper esophagus of our 2.5-year-old patient.

### Case presentation

**Fig. 1:**
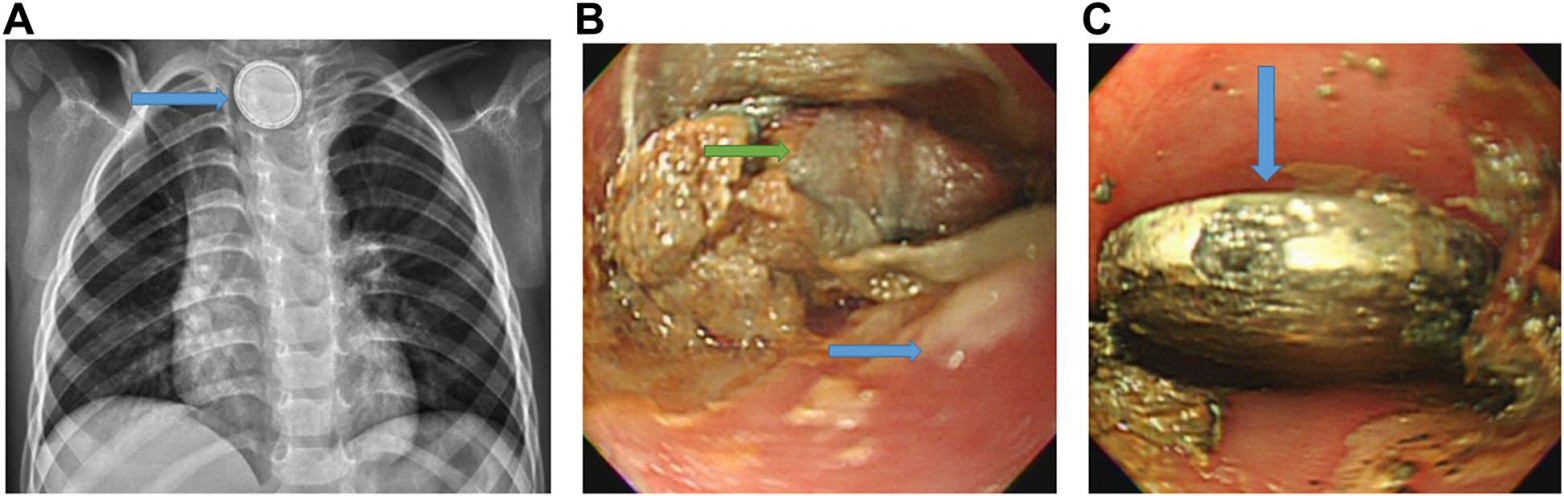
**a** A 21-mm round, foreign body with double ring halo sign (arrow) in the upper esophagus. **b** A round foreign body (green arrow) is covered with a mixture of ingested food and necrotic tissue of the esophagus (blue arrow). **c** The round metal foreign body (arrow) is observed after removing the ingested food

A 2.5-year-old male child was carried to the emergency room of a children’s hospital in central Taiwan. His guardian claimed that he had been previously healthy, but suffered from fever, vomited undigested food, and refused to eat for one day. He had cough and rhinorrhea for more than one week and completed a course of azithromycin. The patient had no hoarseness or drooling when vocalizing. On exam, he was alert and conscious with a normal neurological examination and no unsteady gait. On chest auscultation, he had a normal heart sounds and bilateral rhonchi but no respiratory distress. His abdomen was mildly distended, but soft on palpation. Plain abdominal radiographs were obtained which showed some fecal material within the colon but no other positive findings. As this could not adequately explain the child's severe gastrointestinal symptoms, a chest radiograph was obtained. The chest radiograph showed increased left perihilar and right lower lung infiltration, and a 21-mm round foreign body with a double ring halo was found on the upper esophagus (Fig. 1A). We determined that the boy suffered from esophageal foreign body impaction, possibly from BBI.

**Fig. 2:**
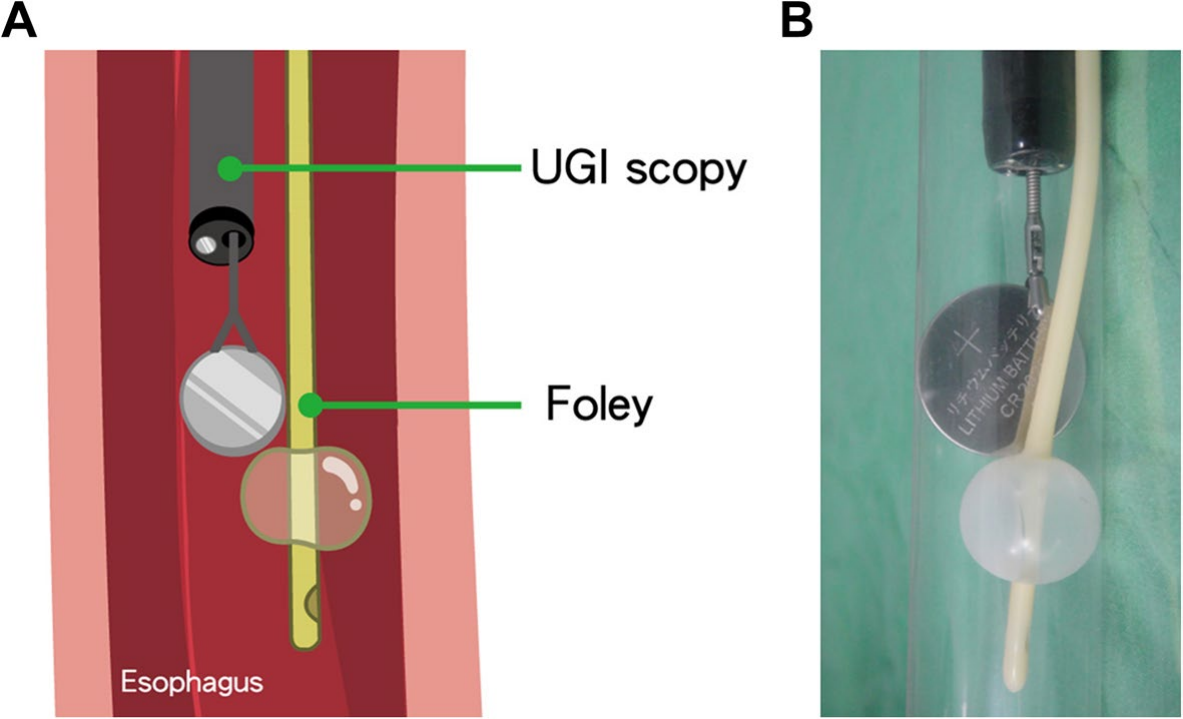
An 8 Fr. Foley catheter is inserted to an area distal to the foreign body, and 2 cc distilled water is infused into the balloon of the Foley catheter. **a** A cartoon illustration. **b** A real photo

Within 2 h of arrival to the emergency room, he was in the operating room undergoing an upper gastrointestinal endoscopic examination (GIF XQ-240, Olympus Corp., Tokyo, Japan) and retrieval procedure. Under direct view on endoscopy (Figs. 1B and 1C), the round foreign body was found covered with a mixture of ingested food and necrotic esophageal tissue. We tried to remove the foreign body with endoscopic alligator forceps many times but failed to retrieve it. A Roth Net retriever (STERIS, Mentor, OH, USA) was then used but also failed since the net could hardly open in the narrow esophageal lumen. Afterward, an 8 Fr. Foley catheter was inserted via the nasal cavity and advanced to an area distal to the foreign body. Once in place, 2 cc distilled water was infused into the balloon of the Foley catheter (Fig. 2). Two operators were needed for this procedure. One of them operated the endoscope, and the other operated the alligator forceps and the Foley. Under the view by endoscope, we were able to stop and adjust the instruments when displacement occurred, either of the instruments themselves or of the button battery. We then pulled out the Foley catheter and simultaneously clamped the button battery with alligator forceps. The button battery was thus secured and removed with simultaneous use of both tools.

**Fig. 3:**
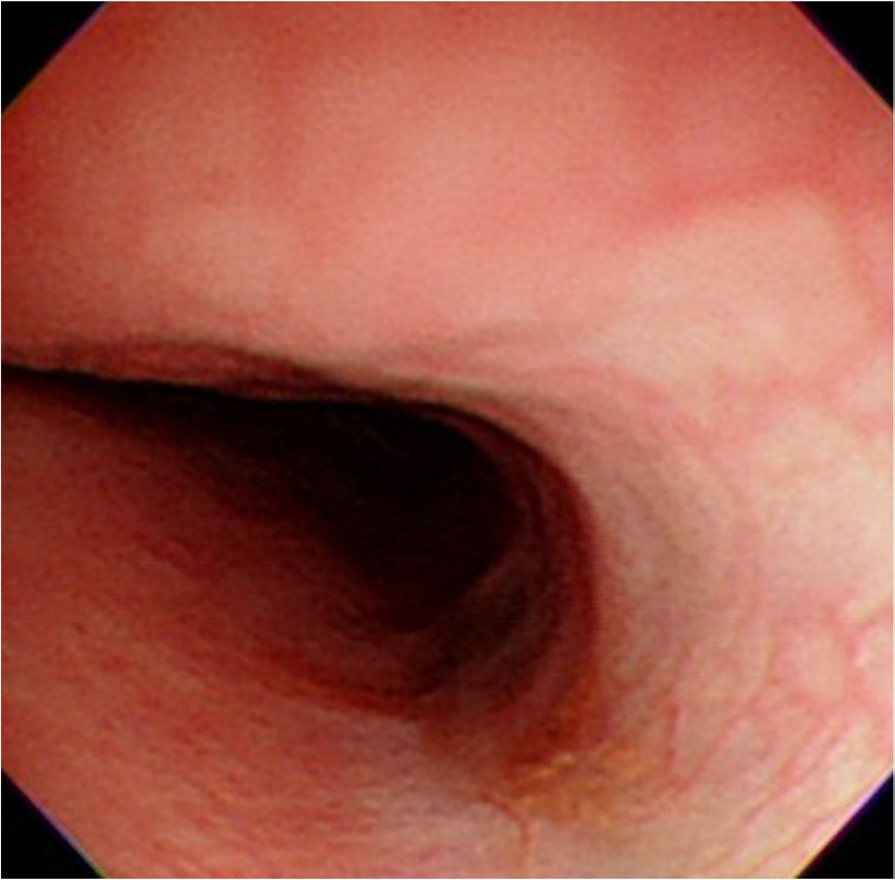
Follow-up endoscopic examination shows healed ulcers and no stricture formation

The endoscopic exam of the area after removal of the button battery showed ulceration with erythematous change in over 50% of the circumference at the impaction area of the upper esophagus. A nasogastric tube was placed in the operating room. He was transferred to an inpatient ward where he was not allowed to orally ingest anything. He received prophylactic antibiotics, an H-2 blocker, and steroids for nine days. The day after surgery, the patient’s follow-up chest X-ray showed no free air in the mediastinum. He started on a clear-liquid diet two days after the procedure. No vomiting or dysphagia was observed, and a soft diet was initiated. After five days of hospitalization, he was discharged with stable vital signs and no difficulty with oral intake. One month later, a follow-up endoscopic examination (Fig. 3) showed healed ulcers and no stricture formation.

## Discussion and conclusions

The esophageal injury from BBI derives from three aspects. First, the external electric current hydrolyzes the fluid content of adjacent tissue and produces hydroxide ions. Second, there is leakage of alkaline compounds. Finally, there is direct compression on the adjacent tissue. Among these, the presence of the hydroxide ion is the major cause of injury in the esophagus [4]. The trauma to the body begins as soon as 15 min after battery ingestion. Severe burns with sequelae may occur within two hours [1, 4]. Unfortunately, the injuries may continue for days to weeks, even after battery removal [3]. Some reported complications include vocal cord paralysis, esophageal perforation, trachea-esophageal fistula, fistula formation of major vessels, and possibly death.

There are no unique symptoms or signs attributable to esophageal battery impaction. The most common symptoms or signs are cough, dysphagia, and vomiting [2, 5], which are common to many other diseases [2]. Because of the progressive nature of the injury, it is impossible to predict the severity of outcomes according to the presenting symptoms and signs [2]. In 2015, the North American Society for Pediatric Gastroenterology, Hepatology & Nutrition Endoscopy Committee recommended that once esophageal button battery impaction is diagnosed or suspected, immediate removal is essential [3].

Because the time of ingestion is often unknown, when an esophageal button battery impaction is confirmed, an endoscopic examination is needed for evaluating the extent of esophageal injury [5]. Given that foreign body ingestions are often unwitnessed, battery impaction must be kept in mind as a possible diagnosis if previously healthy children have a sudden onset of vomiting with undigested food or massive hematemesis [2, 6]. In the present case, the patient presented with a fever, vomiting with undigested food, and refused to eat. Therefore, our pursuit of the disease etiology focused on the gastrointestinal system at first. In such cases, plain abdominal film or abdominal ultrasonography should be performed immediately. If no positive findings correlate with the symptoms, X-ray imaging of the chest should be performed to exclude esophageal foreign body impaction.

For the retrieval methods, the oral-gastric tube with a magnetic tip under fluoroscopy and rigid esophageal endoscopy technique have the highest success rates [7], but each method has its own risks to consider. Rigid esophageal endoscopy may slightly increase the perforation rate [8], although the difference has not been shown to be significant in children [9, 10]. A net retriever is excellent for retrieving smooth objects such as button batteries [6, 11]. However, in a small space such as the esophageal lumen, its effectiveness is reduced [6]. The alligator forceps are proper for retrieving rough-surface objects, but they are difficult to operate with smooth-surface objects, such as button batteries. In addition, adhesions of the objects to soft tissue can decrease the effectiveness of these tools even further. Balloon extraction with fluoroscopy has a lower success rate than other methods [12], possibly due to its low extraction power. Low extraction power means insufficient balloon inflation causing the balloon itself to slide between the gap between the battery and esophageal wall. If over inflated, increasing friction may occur, resulting in esophageal rupture. However, the combination of using endoscopic balloon extraction and forceps retrieval resulted in a synergistic push-and-pull effect. It successfully prevented the battery from relodging in another location during the operation. With the view provided by the endoscope, we could immediately adjust to prevent the battery from sliding off or becoming displaced. Regarding cost, the Foley catheter is cheap and widely available. No matter what methods are chosen, post-removal endoscopic examination is strongly recommended for evaluating the procedure’s success and to determine need for follow-up treatment.

If intermittent vomiting is observed in previously healthy children, lower gastrointestinal etiology is suspected in most cases. However, esophageal problems such as foreign body ingestion should be considered, especially in infants, toddlers, or developmentally delayed children, and excluded after thorough clinical investigation. In the present case, chest X-rays were indicated after considering lower gastrointestinal etiology. When facing a difficult retrieval of an esophageal foreign body, the novel retrieval method described herein may be considered. This new procedure was very effective in removing the esophageal foreign body. When button battery in esophagus was too tight to be removed by the traditional retrieval methods, this procedure was suggested to use. This procedure could be performed at medical institutions. If it fails or esophageal perforation (iatrogenic or spontaneous) occurs, pediatric surgeons could take over immediately.

## Data Availability

Data sharing is not applicable to this article as no datasets were generated or analyzed during the current study.

## Abbreviation

BBI: Button battery ingestion
